# Protocol for a systematic review and meta-analysis of the combination of ezetimibe and statins for hyperlipidemia

**DOI:** 10.1371/journal.pone.0312588

**Published:** 2024-12-23

**Authors:** Tianfu Yang, Weijuan Li, Weiwei Chen, Donghong Zhu, Yuxi Ren, Xiongfeng Huang

**Affiliations:** 1 The First People’s Hospital of Fuzhou, Fuzhou, Jiangxi, China; 2 Fuzhou Medical College, Nanchang University, Fuzhou, Jiangxi, China; 3 Department of Respiratory, The Ninth Hospital of Nanchang, Nanchang, Jiangxi, China; 4 Jiangxi University of Chinese Medicine, Nanchang, Jiangxi, China; Huashan Hospital Fudan University, CHINA

## Abstract

**Introduction:**

Hyperlipidemia is increasingly recognized as a significant global health issue, often associated with conditions such as hypertension, diabetes, and obesity. While statins are frequently prescribed to manage lipid levels, recent studies indicate that reliance solely on statin therapy may present certain disadvantages, including prolonged treatment durations, the potential for drug resistance, and various adverse effects. Research indicates that the combination of ezetimibe and statins demonstrates a favorable therapeutic effect in the management of hyperlipidemia. However, existing studies have not consistently confirmed these benefits, and there is no current meta-analysis available. As a result, we will perform a meta-analysis to assess the effectiveness and safety of the combination of ezetimibe and statins in managing hyperlipidemia, aiming to offer evidence-based medical guidance for clinical practice.

**Methods and analysis:**

The systematic review and meta-analysis will adhere to the PRISMA guidelines for systematic reviews and meta-analyses. We will search for randomized controlled trials that investigate the efficacy and safety of the combination of ezetimibe and statins in treating hyperlipidemia, based on specific criteria. The following electronic databases will be searched by two researchers for relevant records published up to October 1, 2024: Cochrane Central Register of Controlled Trials (CENTRAL) in Cochrane Library, Embase.com, Web of Science, MEDLINE (via PubMed), Wanfang China Database, China National Knowledge Infrastructure (CNKI), Chinese Biomedical Literature Database (CBM) and Chinese Scientific Journal Database (VIP). They will also check references and relevant journals manually. Two independent reviewers will handle screening, data extraction, and quality assessment. Subgroup analysis, sensitivity analysis, and publication bias analysis will be performed to assess consistency and reliability. Review Manager 5.4 will be used for data analysis and synthesis, while the GRADE approach will be employed to evaluate the overall study’s evidence quality.

**Expected results:**

The findings of this systematic review will be shared with various stakeholders who are interested in the combination of ezetimibe and statins for hyperlipidemia. This will offer valuable insights for researchers undertaking future investigations and for clinical practitioners specializing in the treatment of hyperlipidemia.

**Ethics and dissemination:**

This study is based on a secondary analysis of the literature, so ethical review approval is not required. The final report will be published in a peer-reviewed journal.

**Study registration:**

The protocol of the systematic review has been registered on Open Science Framework, with a registration DOI https://doi.org/10.17605/OSF.IO/TEVUY.

## Introduction

Hyperlipidemia has become a modern epidemic, fueled by changes in diet and lifestyle. It is a condition characterized by abnormalities in lipid metabolism within the bloodstream [1]. This condition is primarily manifested by elevated levels of total cholesterol, triglycerides, low-density lipoprotein (LDL), or decreased levels of high-density lipoprotein (HDL) [2–4]. In recent years, with the rapid socioeconomic development and significant changes in people’s lifestyles, the average serum cholesterol levels have gradually increased. Hyperlipidemia is a significant cause of deaths related to cardiovascular disease and has significant economic consequences, posing a major challenge to the worldwide public health system [5–7].

Studies have shown that dyslipidemia is a significant risk factor for cardiovascular diseases [7–11]. Elevated lipid levels can lead to the accumulation of plaque within the arteries, a condition known as atherosclerosis. This can narrow the blood vessels, reducing blood flow and increasing the risk of heart attacks, strokes, and other cardiovascular diseases. Furthermore, hyperlipidemia is also associated with an increased risk of type 2 diabetes, hypertension, and other chronic health conditions. Therefore, the prevention and control of hyperlipidemia, or high blood lipids, are of utmost importance for maintaining overall health.

The management of hyperlipidemia encompasses two primary strategies: lifestyle modifications and pharmacological interventions [12]. Lifestyle changes [13], including dietary adjustments, weight management, and the modification of detrimental habits, constitute the cornerstone of lipid-lowering therapy and are consistently applied throughout the treatment process. In clinical practice, pharmacological options for lipid reduction include statins, fibrates, cholesterol absorption inhibitors, and niacin. Currently, statins are the primary medication used to treat hyperlipidemia [14, 15]. However, they may cause adverse effects like muscle pain and liver damage [16]. Fibrates serve as second-line treatments, but they can raise serum creatinine levels and are linked to risks such as sudden death, pancreatitis, and venous thrombosis [17–19].

Research indicates that the combination of ezetimibe and statins demonstrates a favorable therapeutic effect in the management of hyperlipidemia [20]. Ezetimibe functions as a selective inhibitor of cholesterol absorption, effectively reducing the uptake of dietary and biliary cholesterol by targeting the C-type Class 1 Niemann-Pick C1-like 1 (NPC1L1) protein [21]. This mechanism enhances the synthesis of LDL receptors in the liver, thereby facilitating the metabolism of LDL and subsequently lowering plasma cholesterol levels.

At present, many studies have reported the positive impact of the combination of ezetimibe and statins on patients with hyperlipidemia [22–26], but the results are inconsistent and the quality of the studies varies, which generates some confusion for clinicians and patients. Therefore, it is very necessary to systematically evaluate and conduct a meta-analysis on the intervention of the combination of ezetimibe and statins on hyperlipidemia. This study will systematically evaluate and conduct a meta-analysis on the effectiveness and safety of the combination of ezetimibe and statins in the treatment of hyperlipidemia, aiming to offer evidence-based medical guidance for clinical practice.

## Methods and analysis

### Registration

This protocol was created following the guidelines outlined in the PRISMA-P (Preferred Reporting Items for Systematic Review and Meta-Analysis Protocols) statement [27]. The PRISMA-P checklist is available for reference in S1 File. The study protocol was officially registered in the Open Science Framework (DOI: https://doi.org/10.17605/OSF.IO/TEVUY). We will document any protocol deviations in the final report.

### Criteria for considering studies for this review

#### Types of studies

This study will only include randomized controlled trials (RCTs). To maintain high standards of evidence, trials with inadequate design, duplicate publication, missing important details, issues with randomization or statistical methods, incomplete documentation (both manual and electronic), data anomalies, inconsistencies in baseline data, or missing data will be strictly excluded from our analysis. There are no limitations on language or publication date.

#### Participants

Individuals were diagnosed with hyperlipidemia regardless of their demographic attributes, including age, ethnicity, nationality, or gender.

#### Interventions

The treatment group received a combination of statins and ezetimibe, while the control group was administered statins exclusively. Participants were required to undergo the intervention for a minimum duration of four weeks.

#### Outcomes

The primary outcomes include levels of total cholesterol (TC), triglycerides (TG), low-density lipoprotein cholesterol (LDL-C), high-density lipoprotein cholesterol (HDL-C), and apolipoprotein. Supplementary outcomes encompass adverse events, body mass index (BMI), and waist-to-hip ratio.

### Search methods for identification of studies

We will thoroughly search for all studies that have been published, unpublished or are currently ongoing, without any restrictions on language, location, or publication status. Our search will include databases such as Cochrane Central Register of Controlled Trials (CENTRAL) in Cochrane Library, Embase.com, Web of Science, MEDLINE (via PubMed), Wanfang China Database, China National Knowledge Infrastructure (CNKI), Chinese Biomedical Literature Database (CBM) and Chinese Scientific Journal Database (VIP) from the inception of these databases up to October 1, 2024. The comprehensive search strategy employed for PubMed is presented in Table 1, while the elaborate search strategy utilized for additional databases can be found in S2 File. We will review the reference lists of the studies that are included and any related reviews to find more relevant studies. We will reach out to experts or organizations in the field to gather more information on relevant studies. If needed, we will get in touch with the authors of the included studies to clarify data and obtain additional information.

**Table 1:** Search strategy for PubMed.

Number	Search terms
#1	hyperlipidemia OR hyperlipidemias OR dyslipidemias OR hyperlipidemia OR dyslipidemias OR hyperlipoproteinemia OR hypertriglyceridemia OR hypercholesterolemia OR hypercholesteremia OR high blood lipids OR hyperlipemia OR dyslipidemia [Title/Abstract]
#2	ezetimibe OR statin OR atorvastatin OR simvastatin OR lovastatin OR pravastatin OR rosuvastatin [Title/Abstract]
#3	randomly OR randomized OR RCT OR trials [Title/Abstract]
#4	#1 AND #2 AND #3 [Title/Abstract]

### Selection of studies

**Fig 1:**
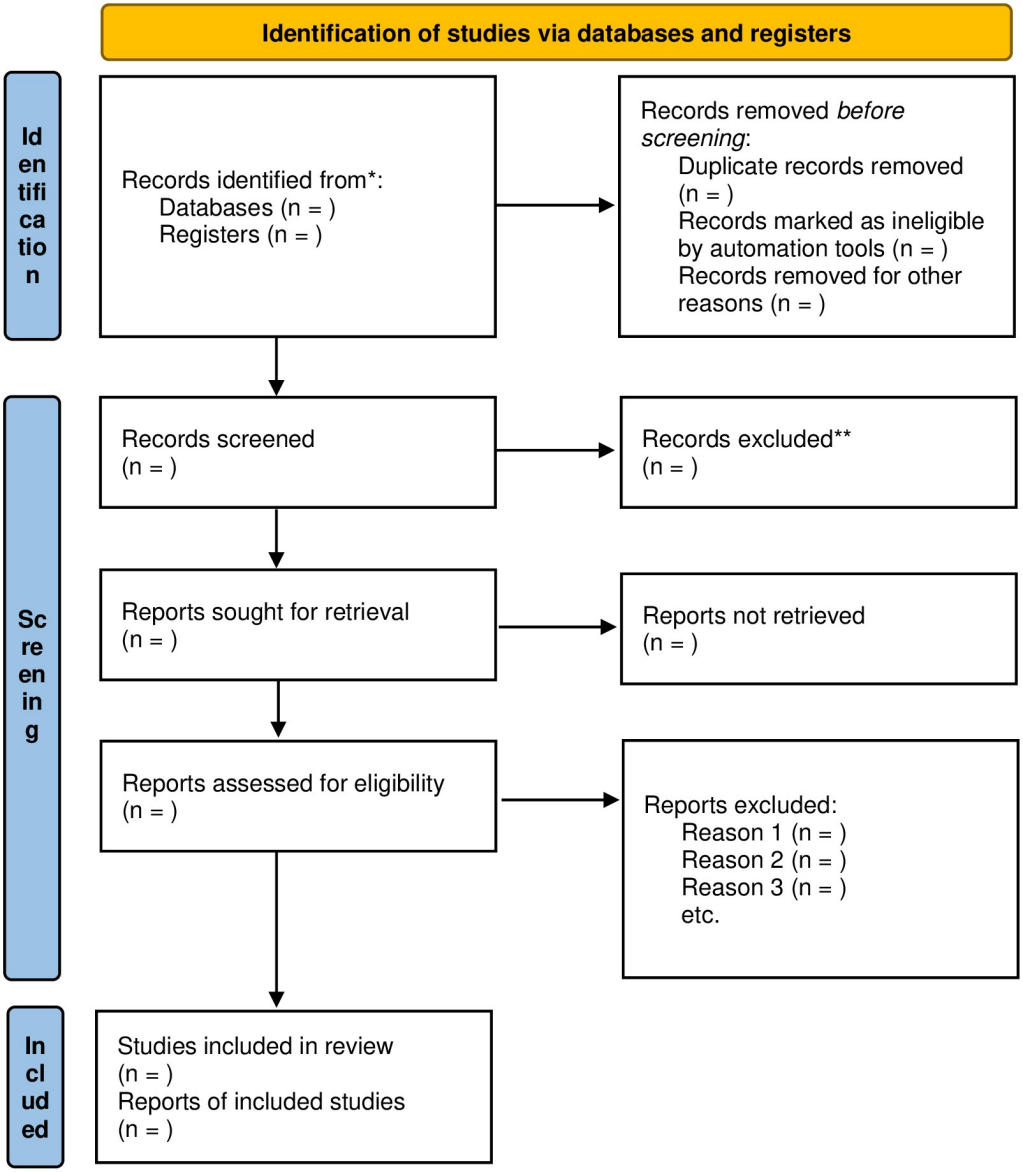
Flow diagram showing the selection process of articles. *Consider, if feasible to do so, reporting the number of records identified from each database or register searched (rather than the total number across all databases/registers). **If automation tools were used, indicate how many records were excluded by a human and how many were excluded by automation tools. *From*: Page MJ, McKenzie JE, Bossuyt PM, Boutron I, Hoffmann TC, Mulrow CD, et al. The PRISMA 2020 statement: an updated guideline for reporting systematic reviews. BMJ 2021;372:n71. doi: 10.1136/bmj.n71. For more information, visit: http://www.prisma-statement.org/.

Two reviewers will independently evaluate the titles and abstracts of retrieved studies for potential inclusion. Full-text reports will be obtained and both reviewers will independently determine the eligibility of the papers. Reasons for excluding studies will be documented. Any disagreements will be resolved through discussion, with a third reviewer consulted if necessary. The selection process will be outlined in a PRISMA flow diagram (Fig 1).

### Data extraction

Two authors will independently extract data using standard forms that include study details (author, country, publication year, language, journal, article title), participant information (sample size, average age, gender distribution), intervention specifics (frequency, duration), comparator details (drug name, dose, frequency, duration), methodological aspects (study design, randomization, allocation, blinding), and outcomes (primary and secondary outcomes). If data are incomplete or unclear, the corresponding author will be contacted for clarification. Any disagreements will be resolved through consensus or by involving a third author.

### Risk of bias assessment

The Cochrane Risk of Bias Tool version 2 [28], created by the Cochrane Collaboration to assess bias in randomized trials, will be utilized to evaluate the bias level in the literature included. This tool addresses seven key areas: random sequence generation, allocation concealment, blinding of participants and staff, blinding of outcome assessment, incomplete outcome data, selective reporting, and other potential sources of bias [29]. We will assess each possible bias as ’high’, ’low’, or ’unclear’ and explain the rationale for this assessment in the bias risk table, citing the study report [30]. Two unbiased reviewers will thoroughly evaluate the bias risk in the chosen research. Any discrepancies between the reviewers will be settled by involving a third independent investigator. RevMan V.5.4 will be utilized to create graphs and detailed summaries illustrating the bias risk assessment.

## Statistical analysis

### Data synthesis

The analysis of data will be conducted using RevMan (V.5.4) software. Risk ratios (RR) with 95% confidence intervals will be utilized for analyzing dichotomous data, while weighted mean difference (WMD) or standardized mean difference (SMD) with a 95% confidence interval will be employed for continuous data. If results are measured on the same assessment tool or scale, WMD will be used; otherwise, SMD will be the preferred method. Statistical significance will be assessed at a significance level of P<0.05 [31, 32].

The researchers will evaluate the presence of diversity within the data determine whether to conduct a meta-analysis and select an appropriate model based on the findings. If there is no substantial diversity found (I^2^≤50%), they will utilize the fixed-effect model [33, 34]. Conversely, if the I^2^ value exceeds 50%, indicating diversity, they will focus on investigating potential sources of clinical, methodological, or statistical diversity. In cases of statistical diversity, a random-effects model will be utilized to combine the data [35, 36]. Narrative analyses will be carried out when it is not possible to conduct a combined data analysis.

### Subgroup analyses

Subgroup analyses will be conducted on the findings to explore potential sources of diversity. These analyses will be carried out if sufficient data are accessible to identify differences in various factors, such as different drugs, risk of bias levels in the included RCTs, participant demographics, sample size, duration of follow-up, treatment frequency, and duration of treatment.

### Sensitivity analyses

In the event deemed suitable, sensitivity analyses will be conducted to ascertain the strength and dependability of the review’s findings of significant outcomes. The leave-one-out method will be employed to gauge the impact of individual studies on the overarching meta-analysis, to identify any undue influence exerted by a specific study. For instance, the exclusion of individual studies will be undertaken to assess its impact on the extent of heterogeneity. This method serves as a systematic approach to scrutinize the contribution of each study to the observed heterogeneity.

### Grading the quality of evidence

The certainty of evidence will be evaluated using the GRADE (Grading of Recommendations Assessment, Development and Evaluation) system, which involves assessing five domains of study limitations (risk of bias, inconsistency, indirectness, imprecision, and publication bias) [37–39]. The quality of evidence will be categorized as ’very low’, ’low’, ’moderate’, or ’high’ based on these criteria. Two reviewers will independently assess the certainty of evidence, with a third reviewer brought in to resolve any disagreements. Justifications for these assessments will be provided and documented for each outcome in the reporting.

### Assessment of reporting biases

In order to assess the likelihood of publication bias, inverted funnel plots will be created. These plots will be utilized for the evaluation of publication bias probability. Additionally, Egger’s test will be employed on meta-analyses that include over 10 trials to investigate the potential presence of publication bias.

### Patient and public involvement

This research will not include patients or the general public in the process of designing, carrying out, reporting, or planning for dissemination.

### Ethics and dissemination

Since the data used in this study are obtained from published research through databases and do not involve direct contact with patients, ethical approval is not required. The research findings will be shared in reputable academic journals.

### Updates to study protocol

If any modifications to the review protocol are necessary, these adjustments will be documented and included as supplementary material alongside the final manuscript, and will also be updated on the OSF register.

## Discussion

Hyperlipidemia is a disorder characterized by disrupted lipid metabolism, which manifests as elevated levels of TC, TG, or LDL-C, and a decrease in HDL-C in the circulatory system. Statins are commonly prescribed pharmaceuticals for regulating lipid profiles in medical practice. Nevertheless, contemporary research has identified drawbacks associated with exclusive dependence on conventional medical approaches, such as extended treatment periods, vulnerability to drug resistance, and possible adverse reactions. In recent years, ezetimibe has garnered increased attention for its therapeutic attributes and promising prospects. Presently, numerous research endeavors have highlighted the beneficial effects of the combination of ezetimibe and statins in individuals with hyperlipidemia; however, the findings exhibit discrepancies and the methodological quality of these studies varies, leading to ambiguity for healthcare providers and patients. Hence, it is imperative to systematically assess and perform a meta-analysis on the impact of the combination of ezetimibe and statins intervention on hyperlipidemia. Nonetheless, there exist certain potential limitations. Variations in drug dosage, treatment regimens, and outcome measures across different studies may contribute to increased heterogeneity, thereby potentially compromising the objectivity of the conclusions drawn.

The GRADE system can provide a standardized method for assessing the quality of evidence and the strength of recommendations. We will carefully assess the certainty of the evidence and reach reliable conclusions by taking into account the five GRADE considerations: publication bias, consistency of effect, imprecision, indirectness, and risk of bias. The certainty of the evidence classified by GRADE can help in clinical decision-making after the conclusion of the systematic review in several ways: Firstly, it can clarify the strength of the recommendation. GRADE rated the strength of the recommendation as ’very low’, ’low’, ’moderate’, or ’high’ based on the certainty of the evidence. Robust recommendations are issued in the presence of a high level of evidence, whereas practice considerations are offered when the evidence is deemed moderate. In instances where the evidence falls below the moderate threshold, it is indicated that the scientific literature lacks sufficient evidence to inform policymakers, clinicians, and patients effectively. This clear recommendation strength helps clinicians make rapid decisions in the face of complex clinical situations. Secondly, it can promote the rational allocation of medical resources. The certainty of the evidence can help decision-makers assess the benefits and risks of different treatments, so as to rationalize the allocation of medical resources. For treatment modalities that demonstrate a high level of evidence certainty and clear advantages, it is advisable to allocate resources toward their promotion and implementation. Conversely, for treatment approaches characterized by low evidence certainty or ambiguous benefits, it is prudent to either conduct further research or exercise caution in their application to prevent the misallocation of resources.

The results of this comprehensive review will be disseminated to a range of stakeholders with an interest in the combination of ezetimibe and statins for hyperlipidemia. These insights will provide significant value to researchers embarking on subsequent studies and healthcare professionals focusing on the management of hyperlipidemia.

## Supporting information

S1 File PRISMA-P (Preferred Reporting Items for Systematic review and Meta-Analysis Protocols) 2015 checklist: Recommended items to address in a systematic review protocol*. (DOC)

S2 File Search strategy. (DOCX)
