# Supplementary material for: Protocol for a systematic review and meta-analysis of the combination of ezetimibe and statins for hyperlipidemia
Source: PLoS One. 2024 Dec 23;19(12):e0312588. doi: 10.1371/journal.pone.0312588 (PMC11666007; doi:10.1371/journal.pone.0312588)
Supplement: S2 File — (DOCX) [file pone.0312588.s002.docx]

**Supplementary file 2. Search strategy**

**1. PubMed**

| **Number** | **Search terms** |
| --- | --- |
| #1 | hyperlipidemia OR hyperlipidemias OR dyslipidemias OR hyperlipidemia OR dyslipidemias OR hyperlipoproteinemia OR hypertriglyceridemia OR hypercholesterolemia OR hypercholesteremia OR high blood lipids OR hyperlipemia OR dyslipidemia [Title/Abstract] |
| #2 | ezetimibe OR statin OR atorvastatin OR simvastatin OR lovastatin OR pravastatin OR rosuvastatin [Title/Abstract] |
| #3 | randomly OR randomized OR RCT OR trials [Title/Abstract] |
| #4 | #1 AND #2 AND #3 [Title/Abstract] |

**2. Embase**

| **Number** | **Search terms** |
| --- | --- |
| #1 | hyperlipidemia:ti,ab,kw OR hyperlipidemias:ti,ab,kw OR dyslipidemias:ti,ab,kw OR hyperlipidemia:ti,ab,kw OR dyslipidemias:ti,ab,kw OR hyperlipoproteinemia:ti,ab,kw OR hypertriglyceridemia:ti,ab,kw OR hypercholesterolemia:ti,ab,kw OR hypercholesteremia OR high blood lipids:ti,ab,kw OR hyperlipemia:ti,ab,kw OR dyslipidemia:ti,ab,kw |
| #2 | ezetimibe:ti,ab,kw OR statin:ti,ab,kw OR atorvastatin:ti,ab,kw OR simvastatin:ti,ab,kw OR lovastatin:ti,ab,kw OR pravastatin:ti,ab,kw OR rosuvastatin:ti,ab,kw |
| #3 | randomly:ti,ab,kw OR randomized:ti,ab,kw OR RCT:ti,ab,kw OR trials:ti,ab,kw |
| #4 | #1 AND #2 AND #3 |

**3. Cochrane Library**

| **Number** | **Search terms** |
| --- | --- |
| #1 | hyperlipidemia:ti,ab,kw OR hyperlipidemias:ti,ab,kw OR dyslipidemias:ti,ab,kw OR hyperlipidemia:ti,ab,kw OR dyslipidemias:ti,ab,kw OR hyperlipoproteinemia:ti,ab,kw OR hypertriglyceridemia:ti,ab,kw OR hypercholesterolemia:ti,ab,kw OR hypercholesteremia OR high blood lipids:ti,ab,kw OR hyperlipemia:ti,ab,kw OR dyslipidemia:ti,ab,kw |
| #2 | ezetimibe:ti,ab,kw OR statin:ti,ab,kw OR atorvastatin:ti,ab,kw OR simvastatin:ti,ab,kw OR lovastatin:ti,ab,kw OR pravastatin:ti,ab,kw OR rosuvastatin:ti,ab,kw |
| #3 | randomly:ti,ab,kw OR randomized:ti,ab,kw OR RCT:ti,ab,kw OR trials:ti,ab,kw |
| #4 | #1 AND #2 AND #3 |

**4. Web of Science**

| **Number** | **Search terms** |
| --- | --- |
| #1 | TS=(hyperlipidemia OR hyperlipidemias OR dyslipidemias OR hyperlipidemia OR dyslipidemias OR hyperlipoproteinemia OR hypertriglyceridemia OR hypercholesterolemia OR hypercholesteremia OR high blood lipids OR hyperlipemia OR dyslipidemia) |
| #2 | TS=( ezetimibe OR statin OR atorvastatin OR simvastatin OR lovastatin OR pravastatin OR rosuvastatin) |
| #3 | TS=(randomly OR randomized OR RCT OR trials) |
| #4 | #1 AND #2 AND #3 |

**5. China National Knowledge Infrastructure (CNKI) (Chinese)**

| **Search terms** |
| --- |
| (主题:“高脂血症”or“血脂异常”or “高胆固醇血症”or“高血脂”) and (主题:  “依折麦布”or“阿托伐他汀”or“辛伐他汀”or“洛伐他汀”or“普伐他汀” or“瑞舒伐他汀”) and (全部:“随机对照试验”or“随机”) |

**6. WANFANG DATA (Chinese)**

| **Search terms** |
| --- |
| (主题:“高脂血症”or“血脂异常”or “高胆固醇血症”or“高血脂”) and (主题: “依折麦布”or“阿托伐他汀”or“辛伐他汀”or“洛伐他汀”or“普伐他汀” or“瑞舒伐他汀”) and (全部:“随机对照试验”or“随机”) |

**7. Chinese biomedical literature service system (SinoMed) (Chinese)**

| **Search terms** |
| --- |
| ("高脂血症" [常用字段:智能] OR "血脂异常" [常用字段:智能] OR "高胆固醇血症" [常用字段:智能] OR "高血脂" [常用字段:智能]) AND ("依折麦布"[全部字段:智能] OR "阿托伐他汀"[全部字段:智能]OR "辛伐他汀"[全部字段:智能] OR "洛伐他汀"[全部字段:智能]OR "普伐他汀"[全部字段:智能]OR "瑞舒伐他汀"[全部字段:智能]) AND ("随机对照试验"[常用字段:智能] OR "随机"[常用字段:智能]) |

**8. VIP database (Chinese)**

| **Search terms** |
| --- |
| (主题:“高脂血症”or“血脂异常”or “高胆固醇血症”or“高血脂”) and (主题: 依折麦布”or“阿托伐他汀”or“辛伐他汀”or“洛伐他汀”or“普伐他汀” or“瑞舒伐他汀”) and (全部:“随机对照试验”or“随机”) |
